# Supplementary material for: The impact of hypoglycemia on quality of life and related outcomes in children and adolescents with type 1 diabetes: A systematic review
Source: PLoS One. 2021 Dec 2;16(12):e0260896. doi: 10.1371/journal.pone.0260896 (PMC8638919; doi:10.1371/journal.pone.0260896)
Supplement: S1 File — (PDF) [file pone.0260896.s001.pdf]

Citation

Frans Pouwer, Jane Speight, Melanie Broadley, Christel Hendrieckx, Hannah Chatwin, Manon Coolen, Kevin Matlock, Mette Valdersdorf Jensen, Anna Cantrell, Mark Clowes, Louise Preston, Anthea Sutton, Helen Woods. The psychosocial impact of hypoglycaemia: a series of systematic reviews. PROSPERO 2020 CRD42020154023 Available from:
https://www.crd.york.ac.uk/prospero/display_record.php?ID=CRD42020154023

Review question

What is the psychosocial impact of hypoglycaemia in people with diabetes and their family members?

Due to the breadth of this question, we will be developing a series of manuscripts based on the results of our systematic search. These manuscripts will focus on different sub-populations (e.g., based on diabetes diagnosis and age), and on psychosocial outcomes related to quality of life.

Searches

A systematic literature research will be constructed around four key concepts:

- Population terms (1): diabetes (type 1 or type 2)

- Population terms (2): hypoglyc(a)emia

- Outcomes: psychosocial impact (quality of life).

- Study types: empirical qualitative and quantitative studies and systematic reviews

The following electronic databases will be searched: MEDLINE, PsycINFO, CINAHL, and the Cochrane Library. Web of Science will be used for forward and backward-chaining (citation searching). No language or date restrictions will be applied. The PRESS checklist will be used to review the electronic search strategy.

The aim of the systematic review is to examine psychosocial impact and quality of life outcomes broadly. To minimize the bias inherent in deciding on outcome terms a priori, the review team will employ the following strategies:

- Use of broad search terms (e.g., “quality of life,” “subjective wellbeing”)

- Searching PsycINFO for “diabetes” and “hypoglyc(a)emia” without outcome terms, based on the assumption that all records are likely to be from a psychological perspective

- Forward and backward-chaining (citation searches)

Types of study to be included

We will examine data from randomized controlled trials, observational studies, and systematic reviews to determine the current evidence base for the psychosocial impact of hypoglycaemia in people with diabetes and their families. Qualitative studies (e.g., interviews, focus groups) will be included in the search and these studies will be synthesized if the final number of records is manageable and if synthesis is feasible.

Condition or domain being studied

People with diabetes (type 1 and type 2) and their family members, psychosocial impact of hypoglycaemia (low blood glucose levels), quality of life.

Participants/population

People with diabetes (type 1 or type 2) and their family members. Five different sub-populations will be

explored in separate review papers; namely, 1) children & adolescents with type 1 diabetes, 2) adults with type 1 diabetes, 3) adults with type 2 diabetes, 4) parents/caregivers of children with type 1 diabetes, and 5) family members of adults with type 1 or type 2 diabetes.

Intervention(s), exposure(s)

The systematic reviews focus on exposure to hypoglycaemia (low blood glucose).

Comparator(s)/control

We will compare those who have experienced hypoglycaemia to those who have experienced:1) no hypoglycaemia, or 2) less frequent or less severe hypoglycaemia. These comparisons will be dependent on how hypoglycaemia was defined and measured in the identified studies.

Main outcome(s)

Scores on measures of quality of life (QoL), subjective/psychological well-being, level of independence, social relationships, finances, sleep, daytime functioning, health status, mood (e.g., depression and anxiety symptoms), cognitive function (e.g., attention, concentration, memory) and academic performance. We are also interested in diabetes-specific psychosocial outcomes, so we will include scores on measures of fear of hypoglycemia and diabetes distress as outcomes.

Measures of effect

Not applicable.

Additional outcome(s)

None.

Measures of effect

Not applicable.

Data extraction (selection and coding)

Titles and abstracts will be screened with reference to the inclusion criteria. Abstract selection will be undertaken by 2-3 reviewers, depending on the number initially identified. An initial 100 references will be screened by all reviewers to ensure accurate interpretation of inclusion criteria. A proportion (at least 10%) of the abstracts will be double-screened by an additional reviewer as a further consistency check. Where queries emerge, they will be discussed amongst the three reviewers until consensus is reached. Any outstanding queries will be resolved by discussion with internal or Hypo-RESOLVE topic experts. Reporting of the search process will follow the PRISMA standards.

Methodological case study (EPPI reviewer): In addition to traditional screening, the Information Resources Group at ScHARR will run a methodological case study of EPPI-Reviewer 4 software. This program features text mining and machine learning functionality and, after a proportion of records have been sifted by hand by the review team, is able to prioritise the remainder and predict the value of continuing to hand sift these records using machine learning techniques. We propose to evaluate the accuracy of EPPI-Reviewer predictions by comparing them with judgements of the review team.

Data extraction for each included full-text article will be undertaken by one reviewer and all data extractions will be checked by a second reviewer. To ensure all relevant study information is extracted and that all reviewers interpret the data extraction criteria accurately, 5 candidate papers will be extracted by all team members. The data to be extracted include: reference information, information on participants (type of diabetes, sample size, age, gender, and other demographic characteristics), information on hypoglycaemia, study design, data collection and analyses procedures, and the measures and outcomes related to hypoglycaemia, psychosocial measures, and quality of life measures.

Risk of bias (quality) assessment

Article quality will be assessed using the Joanna Briggs Institute Critical Appraisal tools.

To ensure quality of searches and extraction methods:

- An experienced team will be responsible for the searching, selection, and data extraction.

- The scoping and full searches will be peer reviewed using the PRESS 2015 Evidence Based Checklist

- At abstract and full-text selection stages, a random sample (100 records) will be screened by all reviewers. Abstract selection will be undertaken by 2-3 reviewers, with an additional reviewer checking at least 10% of records from each reviewer. A Kappa statistic will be calculated and reported to assess and ensure inter-rater reliability

- Where queries emerge, they will be discussed by the all reviewers in order to reach a consensus, or with the wider team of experts if required

- The data extraction method will be quality assured by a methodological expert, and candidate papers will be extracted by all reviewers to ensure consistency and completeness.

Strategy for data synthesis

Data will be synthesized separately for the five sub-population groups outlined above, resulting in five separate manuscripts. The approach to data synthesis across the five reviews will differ according to the characteristics of the included studies. Included qualitative studies will be synthesized following established methodology outlined by Thomas and Harden (2008) in their paper *"Methods for the thematic synthesis of qualitative research in systematic reviews."* For included quantitative studies, meta-analysis may not be feasible. Valentine, Pigott, and Rothstein (2010) recommend the inclusion of results from at least 25 studies to obtain adequate statistical power when examining moderate to small fixed effects, which may not be possible given the anticipated diversity in the definition and measurement of hypoglycaemia, and a diverse range of QoL outcomes in the included studies. As such, narrative synthesis (using the SWiM guidelines) will likely be used for quantitative studies. These syntheses will be structured according to study designs, definition/measures of hypoglycaemia, outcome types and measures, or a combination of the three. All reported relationships between hypoglycaemia and QoL outcomes will be included in the synthesis, regardless of whether these are reported in text (e.g., “no significant relationship was found between hypoglycaemia and depressive symptoms”) as descriptive statistics (e.g., % of participants experiencing work problems after hypoglycaemia) or as inferential statistics (e.g., regression analysis exploring relationships between frequency of hypoglycaemia and QoL scores) with effect sizes. Additionally, we will be considering covariates/confounding variables, where reported, in these relationships.

Analysis of subgroups or subsets

In addition to the population sub-groups being explored in separate systematic review manuscripts, further sub-group analyses may be undertaken within each of these populations.

If the data allow, subset analyses may also be undertaken for:

- Demographic groups (e.g., age, gender, socioeconomic or ethnic background)

- Types of hypoglycaemia (i.e., self-treated versus severe)

- Groups with different hypoglycaemia awareness status (i.e., impaired versus retained/non-impaired)

Contact details for further information

Melanie Broadley
mbroadley@health.sdu.dk

Organisational affiliation of the review

Department of Psychology, University of Southern Denmark, Odense, Denmark

Review team members and their organisational affiliations

Frans Pouwer. Department of Psychology, University of Southern Denmark, Odense, Denmark; STENO Diabetes Center Odense, Odense, Denmark; School of Psychology, Deakin University, Geelong, Australia
Professor Jane Speight. Australian Centre for Behavioural Research in Diabetes (ACBRD), Melbourne,

Australia; School of Psychology, Deakin University, Geelong, Australia
Melanie Broadley. Department of Psychology, University of Southern Denmark, Odense, Denmark
Christel Hendrieckx. Australian Centre for Behavioural Research in Diabetes (ACBRD), Melbourne, Australia; School of Psychology, Deakin University, Geelong, Australia
Ms Hannah Chatwin. Department of Psychology, University of Southern Denmark, Odense, Denmark
Ms Manon Coolen. Department of Psychology, University of Southern Denmark, Odense, Denmark
Mr Kevin Matlock. Department of Psychology, University of Southern Denmark, Odense, Denmark
Ms Mette Valdersdorf Jensen. Department of Psychology, University of Southern Denmark, Odense, Denmark
Anna Cantrell. Information Resources Group, School of Health and Related Research, University of Sheffield, United Kingdom
Mark Clowes. Information Resources Group, School of Health and Related Research, University of Sheffield, United Kingdom
Louise Preston. Information Resources Group, School of Health and Related Research, University of Sheffield, United Kingdom
Anthea Sutton. Information Resources Group, School of Health and Related Research, University of Sheffield, United Kingdom
Helen Woods. Information Resources Group, School of Health and Related Research, University of Sheffield, United Kingdom

Type and method of review
Systematic review

Anticipated or actual start date
12 October 2019

Anticipated completion date
31 May 2020

Funding sources/sponsors
This review is part of the project: Hypoglycaemia - REdefining SOLutions for better liVEs (Hypo-RESOLVE). This project has received funding from the Innovative Medicines Initiative 2 Joint Undertaking (JU) under grant agreement No 777460. The JU receives support from the European Union’s Horizon 2020 research and innovation programme and EFPIA and T1D Exchange, JDRF, International Diabetes Federation (IDF), The Leona M. and Harry B. Helmsley Charitable Trust.

Conflicts of interest

Language
English

Country
Australia, Denmark, England

Stage of review
Review Completed published

Details of final report/publication(s) or preprints if available
First publication: Chatwin H, Broadley M, Speight J, Cantrell A, Sutton A, Heller S, de Galan B, Hendrieckx C, Pouwer F; Hypo-RESOLVE Consortium. The Impact of Hypoglycaemia on Quality of Life Outcomes Among Adults with Type 1 Diabetes: A Systematic Review. Diabetes Res Clin Pract. 2021 Mar 12:108752. doi: 10.1016/j.diabres.2021.108752. Epub ahead of print. PMID: 33722700.

Subject index terms status
Subject indexing assigned by CRD

Subject index terms
Humans; Hypoglycemia

Date of registration in PROSPERO
30 March 2020

Date of first submission
23 October 2019

Stage of review at time of this submission

Stage	Started	Completed
Preliminary searches	Yes	Yes
Piloting of the study selection process	Yes	Yes
Formal screening of search results against eligibility criteria	Yes	Yes
Data extraction	Yes	Yes
Risk of bias (quality) assessment	Yes	Yes
Data analysis	Yes	Yes

The record owner confirms that the information they have supplied for this submission is accurate and complete and they understand that deliberate provision of inaccurate information or omission of data may be construed as scientific misconduct.
The record owner confirms that they will update the status of the review when it is completed and will add publication details in due course.

Versions
30 March 2020
19 March 2021
