# Supplementary material for: The impact of hypoglycemia on quality of life and related outcomes in children and adolescents with type 1 diabetes: A systematic review
Source: PLoS One. 2021 Dec 2;16(12):e0260896. doi: 10.1371/journal.pone.0260896 (PMC8638919; doi:10.1371/journal.pone.0260896)
Supplement: S2 File — (DOCX) [file pone.0260896.s002.docx]

# **S2 Appendix**

**Full search strategy**

Database: Ovid MEDLINE(R) and Epub Ahead of Print, In-Process & Other Non-Indexed Citations, Daily and Versions(R) <1946 to August 20, 2019>

Search Strategy:

--------------------------------------------------------------------------------

1     exp Diabetes Mellitus, Type 1/

2     (("typ* 1" or "typ* I") adj2 diabet*).tw.

3     (IDDM or T1DM or T1D).tw.

4     (("insulin* depend*" or "insulin depend*") not ("non-insulin* depend*" or "noninsulin depend*")).tw.

5     1 or 2 or 3 or 4

6     exp Diabetes Mellitus, Type 2/

7     ("non-insulin* depend*" or "noninsulin depend*").tw.

8     (("typ* 2" or "typ* II") adj2 diabet*).tw.

9     (NIDDM or T2DM or T2D).tw.

10     or/6-9

11     exp Hypoglycemia/ or Blood Glucose Self-Monitoring/

12     (hypoglycemi* or hypoglycaemi* or hypo-glycemi* or hypo-glycaemi* or low blood sugar or low blood glucose or blood glucose monitor*).mp.

13     11 or 12

14     5 and 13 **[T1DM + hypo]**

15     10 and 13 **[T2DM + hypo]**

16     ((psychological or psychosocial or psycho-social) adj3 outcome*).mp. [mp=title, abstract, original title, name of substance word, subject heading word, floating sub-heading word, keyword heading word, organism supplementary concept word, protocol supplementary concept word, rare disease supplementary concept word, unique identifier, synonyms]

17     ("level of independence" or self-efficacy or self-esteem or resilien* or ((social or friend* or marital or partner* or husband* or wife* or spous* or family or familial or families) adj3 relationship*) or social* isolat* or finances or sleep or "daytime functioning" or "cognitive function*" or productivity or (work adj2 absen*) or absenteeism or presenteeism or memory or mood or depress* or anxi* or ((fear or afraid or worr* or distress* or stigma* or impact*) adj3 (hypoglycaemi* or hypoglycemi*)) or "diabetes distress" or "diabetes stigma" or "diabetes burnout" or "psychological conflict").mp.

18     ("care needs" adj3 (express* or perception* or perspective* or judge* or (patient* adj2 view*) or "own assessment*")).mp.

19     Quality of Life/

20     quality of life.mp.

21     (hql or hqol or h qol or hrqol or hr qol).ti,ab,kf.

22     (life satisfaction or wellbeing or well-being).mp.

23     16 or 17 or 18 or 19 or 20 or 21 or 22 **[outcome / QoL terms]**

24     randomized controlled trial.pt. or randomized.mp. or placebo.mp.
**[McMaster therapy filter]**

25     meta analysis.mp,pt. or review.pt. or search:.tw. **[McMaster SR filter]**

26     ((("semi-structured" or semistructured or unstructured or informal or "in-depth" or indepth or "face-to-face" or structured or guide) adj3 (interview* or discussion* or questionnaire*)) or (focus group* or qualitative or ethnograph* or fieldwork or "field work" or "key informant")).ti,ab. or interviews as topic/ or focus groups/ or narration/ or qualitative research/

**[University of Texas qualitative filter]**

27     Epidemiologic studies/ or exp case control studies/ or exp cohort studies/ or Cross-sectional studies/

28     (Case control or cohort analy$).tw.

29     (longitudinal or retrospective or cross sectional).tw.

30     (cohort adj (study or studies)).tw.

31     (Follow up adj (study or studies)).tw.

32     (observational adj (study or studies)).tw.

33     or/27-32 **[SIGN Observational study filter]**

34     (exp child/ not exp adult/) or (child* or adolescen* or teen* or schoolchild* or infant* or paediatric or pediatric).ti.

35     or/24-32 **[ALL eligible study types]**

36     (14 or 15) and 23 and 35

37     14 and 23 and 34 and 35 **[Rev 1: T1DM + hypo + children]**

38     (14 and 23 and 35) not 34 **[Rev 2: T1DM + hypo + adults]**

39     (15 and 23 and 35) not 34 **[Rev 3: T2DM + hypo + adults]**

40     (parent* or carer* or caregiver* or father* or mother* or guardian*).mp. [mp=title, abstract, original title, name of substance word, subject heading word, floating sub-heading word, keyword heading word, organism supplementary concept word, protocol supplementary concept word, rare disease supplementary concept word, unique identifier, synonyms]

41     37 and 40 **[Rev 4: parents of children with T1DM]**

42     ((14 or 15) and 23 and 35) not 34

43     (family or families or spous* or husband* or wife or wives or partner* or son or sons or daughter* or children).mp. [mp=title, abstract, original title, name of substance word, subject heading word, floating sub-heading word, keyword heading word, organism supplementary concept word, protocol supplementary concept word, rare disease supplementary concept word, unique identifier, synonyms]

44     42 and 43 **[Rev 5: families of adults with T1DM or T2DM]**

45     37 or 38 or 39 or 41 or 44 **[Total – all reviews]**
