# Supplementary material for: The impact of hypoglycemia on quality of life and related outcomes in children and adolescents with type 1 diabetes: A systematic review
Source: PLoS One. 2021 Dec 2;16(12):e0260896. doi: 10.1371/journal.pone.0260896 (PMC8638919; doi:10.1371/journal.pone.0260896)
Supplement: S1 Table — (DOCX) [file pone.0260896.s003.docx]

| **Authors** | **Title** | **Year** | **Reason for exclusion** |
| --- | --- | --- | --- |
| Abo-el-Asrar Mohamed ; Andrawes Nevine G; Rabie Menan A; El-Gabry Dina Aly; Khalifa Abdel-Gawad ; El-Sherif Mariam ; Aziz Karim Abdel; | Cognitive functions in children and adolescents with early-onset diabetes mellitus in Egypt | 2018 | Outcomes were cognitive functioning |
| Abraham M B; Nicholas J A; Smith G J; Fairchild J M; King B R; Ambler G R; Cameron F J; Davis E A; Jones T W; Group Plgm Study; | Reduction in Hypoglycemia With the Predictive Low-Glucose Management System: A Long-term Randomized Controlled Trial in Adolescents With Type 1 Diabetes | 2018 | Relationship between hypoglycemia and outcome not assessed |
| Ambler G R; Fairchild J ; Craig M E; Cameron F J; | Contemporary Australian outcomes in childhood and adolescent type 1 diabetes: 10 years post the Diabetes Control and Complications Trial | 2006 | Design not quantitative |
| Bade-White P A; Obrzut J E; | The neurocognitive effects of type 1 diabetes mellitus in children and young adults with and without hypoglycemia | 2009 | Outcomes were cognitive functioning |
| Barnard K D; Wysocki T ; Ully V ; Mader J K; Pieber T R; Thabit H ; Tauschmann M ; Leelarathna L ; Hartnell S ; Acerini C L; Wilinska M E; Dellweg S ; Benesch C ; Arnolds S ; Holzer M ; Kojzar H ; Campbell F ; Yong J ; Pichierri J ; Hindmarsh P ; Heinemann L ; Evans M L; Hovorka R ; | Closing the Loop in Adults, Children and Adolescents With Suboptimally Controlled Type 1 Diabetes Under Free Living Conditions: A Psychosocial Substudy | 2017 | Relationship between hypoglycemia and outcome not assessed |
| Barrio Castellanos ; R ; | Long-acting insulin analogues (insulin glargine or determir) and continuous subcutaneous insulin infusion in the treatment of type 1 diabetes mellitus in the paediatric population | 2005 | Design not quantitative |
| Bohme P ; Bertin E ; Cosson E ; Chevalier N ; group Geode ; | Fear of hypoglycaemia in patients with type 1 diabetes: do patients and diabetologists feel the same way? | 2013 | Other outcomes |
| Brazeau Anne-Sophie ; Nakhla Meranda ; Wright Michael ; Henderson Melanie ; Panagiotopoulos Constadina ; Pacaud Daniele ; Kearns Patricia ; Rahme Elham ; Da Costa ; Deborah ; Dasgupta Kaberi ; | Stigma and its association with glycemic control and hypoglycemia in adolescents and young adults with type 1 diabetes: Cross-sectional study | 2018 | Population not young people with type 1 diabetes |
| Buczkowska E O; Dworzecki T ; Wiedermann G ; Malanowicz B ; Szot D ; | [Continuous subcutaneous insulin infusion (CSII) in treating young diabetic patients] | 2005 | Not in English |
| Burckhardt M A; Abraham M B; Mountain J ; Coenen D ; Paniora J ; Clapin H ; Jones T W; Davis E A; | Improvement in sensosocial outcomes in children with type 1 diabetes and their parents following subsidy for continuous glucose monitoring | 2019 | Relationship between hypoglycemia and outcome not assessed |
| Cai R A; Holt R I. G; Casdagli L ; Viner R M; Thompson R ; Barnard K ; Christie D ; | Development of an acceptable and feasible self-management group for children, young people and families living with Type 1 diabetes | 2017 | Relationship between hypoglycemia and outcome not assessed |
| Castensoe-Seidenfaden P ; Teilmann G ; Kensing F ; Hommel E ; Olsen B S; Husted G R; | Isolated thoughts and feelings and unsolved concerns: adolescents' and parents' perspectives on living with type 1 diabetes - a qualitative study using visual storytelling | 2017 | Design not quantitative |
| Cemeroglu A P; Stone R ; Kleis L ; Racine M S; Postellon D C; Wood M A; | Use of a real-time continuous glucose monitoring system in children and young adults on insulin pump therapy: patients' and caregivers' perception of benefit | 2010 | Relationship between hypoglycemia and outcome not assessed |
| Charron-Prochownik D ; | Social support, chronic stress, and health outcomes in adolescents with diabetes | 1991 | Not published after 2000 |
| Christie D ; Thompson R ; Sawtell M ; Allen E ; Cairns J ; Smith F ; Jamieson E ; Hargreaves K ; Ingold A ; Brooks L ; Wiggins M ; Oliver S ; Jones R ; Elbourne D ; Santos A ; Wong I C; O'Neil S ; Strange V ; Hindmarsh P ; Annan F ; Viner R M; | Effectiveness of a structured educational intervention using psychological delivery methods in children and adolescents with poorly controlled type 1 diabetes: a cluster-randomized controlled trial of the CASCADE intervention | 2016 | Relationship between hypoglycemia and outcome not assessed |
| Christie D ; Thompson R ; Sawtell M ; Allen E ; Cairns J ; Smith F ; Jamieson E ; Hargreaves K ; Ingold A ; Brooks L ; Wiggins M ; Oliver S ; Jones R ; Elbourne D ; Santos A ; Wong I C; O'Neill S ; Strange V ; Hindmarsh P ; Annan F ; Viner R ; | Structured, intensive education maximising engagement, motivation and long-term change for children and young people with diabetes: a cluster randomised controlled trial with integral process and economic evaluation - the CASCADE study | 2014 | Relationship between hypoglycemia and outcome not assessed |
| Clapin H ; Hop L ; Ritchie E ; Jayabalan R ; Evans M ; Browne-Cooper K ; Peter S ; Vine J ; Jones T W; Davis E A; | Home-based vs inpatient education for children newly diagnosed with type 1 diabetes | 2016 | Relationship between hypoglycemia and outcome not assessed |
| Dammacco F ; Torelli C ; Frezza E ; Piccinno E ; Tansella F ; | Problems of hypoglycemia arising in children and adolescents with insulin-dependent diabetes mellitus. The Diabetes Study Group of The Italian Society of Pediatric Endocrinology & Diabetes | 1998 | Not published after 2000 |
| Deeb A ; | Challenges of Diabetes Management in Toddlers | 2017 | Design not quantitative |
| Dever M ; | Screening for Depression in Adolescents With Diabetes | 2016 | No exposure of hypoglycemia |
| Dorchy H ; | [Rational use of insulin analogues in the treatment of type 1 diabetic children and adolescents: personal experience] | 2006 | Not in English |
| Forsander G ; Bogelund M ; Haas J ; Samuelsson U ; | Adolescent life with diabetes-Gender matters for level of distress. Experiences from the national TODS study | 2017 | No exposure of hypoglycemia |
| Fox C ; | The insulin-dependent patient: perceptions and preferences | 1995 | Not published after 2000 |
| Fox L A; Buckloh L M; Smith S D; Wysocki T ; Mauras N ; | A randomized controlled trial of insulin pump therapy in young children with type 1 diabetes | 2005 | Relationship between hypoglycemia and outcome not assessed |
| Frontino G ; Bonfanti R ; Scaramuzza A ; Rabbone I ; Meschi F ; Rigamonti A ; Battaglino R ; Favalli V ; Bonura C ; Sicignano S ; Gioia E ; Zuccotti G V; Cerutti F ; Chiumello G ; | Sensor-augmented pump therapy in very young children with type 1 diabetes: an efficacy and feasibility observational study | 2012 | Relationship between hypoglycemia and outcome not assessed |
| Gabele A ; Budde J ; Spichiger E ; | [Transition from childhood to adolescence-a qualitative study on experiences of adolescents with type 1 diabetes mellitus and their mothers] | 2015 | Not in English |
| Gandrud L ; Altan A ; Buzinec P ; Hemphill J ; Chatterton J ; Kelley T ; Vojta D ; | Intensive remote monitoring versus conventional care in type 1 diabetes: A randomized controlled trial | 2018 | Relationship between hypoglycemia and outcome not assessed |
| Garza K P; Jedraszko A ; Weil L E. G; Naranjo D ; Barnard K D; Laffel L M. B; Hood K K; Weissberg-Benchell J ; | Automated Insulin Delivery Systems: Hopes and Expectations of Family Members | 2018 | Design not quantitative |
| Gillibrand Rachel ; Stevenson Jim ; | The extended health belief model applied to the experience of diabetes in young people | 2006 | Relationship between hypoglycemia and outcome not assessed |
| Golden M P; Ingersoll G M; Brack C J; Russell B A; Wright J C; Huberty T J; | Longitudinal relationship of asymptomatic hypoglycemia to cognitive function in IDDM | 1989 | Not published after 2000 |
| Gonder-Frederick L ; Zrebiec J ; Bauchowitz A ; Lee J ; Cox D ; Ritterband L ; Kovatchev B ; Clarke W ; | Detection of hypoglycemia by children with type 1 diabetes 6 to 11 years of age and their parents: a field study | 2008 | Relationship between hypoglycemia and outcome not assessed |
| Grey M ; Boland E A; Davidson M ; Li J ; Tamborlane W V; | Coping skills training for youth with diabetes mellitus has long-lasting effects on metabolic control and quality of life | 2000 | Relationship between hypoglycemia and outcome not assessed |
| Hanna K M; Weaver M T; Stump T E; Dimeglio L A; Miller A R; Crowder S ; Fortenberry J D; | Initial findings: primary diabetes care responsibility among emerging adults with type 1 diabetes post high school and move out of parental home | 2013 | No exposure of hypoglycemia |
| Hanna Kathleen M; Weaver Michael T; Stump Timothy E; Fortenberry J ; DiMeglio Linda A; | The relationship of worry about hypoglycemia with diabetes-specific and typical youth behavior among emerging adults with type 1 diabetes | 2014 | No exposure of hypoglycemia |
| Hanna K M; Weaver M T; Stump T E; Slaven J E; Fortenberry J D; DiMeglio L A; | Readiness for living independently among emerging adults with type 1 diabetes | 2013 | Relationship between hypoglycemia and outcome not assessed |
| Hannonen Riitta ; Tupola Sarimari ; Ahonen Timo ; Riikonen Raili ; | Neurocognitive functioning in children with type-1 diabetes with and without episodes of severe hypoglycaemia | 2003 | Outcomes were cognitive functioning |
| Hannosen Riitta ; Komulainen Jorma ; Riikonen Raili ; Ahonen Timo ; Eklund Kenneth ; Tolvanen Asko ; Koskinen Paivi ; Nuuja Anja ; | Academic skills in children with early-onset type 1 diabetes: The effects of diabetes-related risk factors | 2012 | Outcomes were cognitive functioning |
| He J ; Li S ; Liu F ; Zheng H ; Yan X ; Xie Y ; Li X ; Zhou Z ; Zhu X ; | Glycemic control is related to cognitive dysfunction in Chinese children with type 1 diabetes mellitus | 2018 | Outcomes were cognitive functioning |
| He J ; Ryder A G; Li S ; Liu W ; Zhu X ; | Glycemic extremes are related to cognitive dysfunction in children with type 1 diabetes: A meta-analysis | 2018 | Outcomes were cognitive functioning |
| Hernandez C A; Bradish G I; Laschinger H K. S; Rodger N W; Rybansky S I; | Self-awareness work in Type 1 diabetes: traversing experience and negotiating collaboration | 1997 | Not published after 2000 |
| Hershey T ; Lillie R ; Sadler M ; White N H; | The effect of severe hypoglycaemia on cognition in children and adolescents with type 1 diabetes mellitus | 2002 | Outcomes were cognitive functioning |
| Hershey T ; Lillie R ; Sadler M ; White N H; | Severe hypoglycemia and long-term spatial memory in children with type 1 diabetes mellitus: a retrospective study | 2003 | Outcomes were cognitive functioning |
| Hershey T ; Lillie R ; Sadler M ; White N H; | A prospective study of severe hypoglycemia and long-term spatial memory in children with type 1 diabetes | 2004 | Outcomes were cognitive functioning |
| Hommel E ; Olsen B ; Battelino T ; Conget I ; Schutz-Fuhrmann I ; Hoogma R ; Schierloh U ; Sulli N ; Gough H ; Castaneda J ; de Portu ; S ; Bolinder J ; Group Switch Study; | Impact of continuous glucose monitoring on quality of life, treatment satisfaction, and use of medical care resources: analyses from the SWITCH study | 2014 | Relationship between hypoglycemia and outcome not assessed |
| Hood K K; Huestis S ; Maher A ; Butler D ; Volkening L ; Laffel L M. B; | Depressive symptoms in children and adolescents with type 1 diabetes: association with diabetes-specific characteristics | 2006 | No exposure of hypoglycemia |
| Jabbour G ; Henderson M ; Mathieu M E; | Barriers to Active Lifestyles in Children with Type 1 Diabetes | 2016 | Design not quantitative |
| Jaser S S; | Psychological problems in adolescents with diabetes | 2010 | Design not quantitative |
| Jaser Sarah S; Foster Nicole C; Nelson Bryce A; Kittelsrud Julie M; DiMeglio Linda A; Quinn Maryanne ; Willi Steven M; Simmons Jill H; | Sleep in children with type 1 diabetes and their parents in the T1D Exchange | 2017 | Population not young people with type 1 diabetes |
| Jezequel C ; de Kerdanet ; M ; Girre M A; | [Hypoglycemia provoked by clandestine injections of insulin in the diabetic child] | 1993 | Not published after 2000 |
| Kakleas K ; Kandyla B ; Karayianni C ; Karavanaki K ; | Psychosocial problems in adolescents with type 1 diabetes mellitus | 2009 | Design not quantitative |
| Kent S ; Chen R ; Kumar A ; Holmes C ; | Individual growth curve modeling of specific risk factors and memory in youth with type 1 diabetes: an accelerated longitudinal design | 2010 | Outcomes were cognitive functioning |
| Kent Sheryl Joan; | Specific risk factors and memory over time in youth with type 1 diabetes | 2008 | Outcomes were cognitive functioning |
| King K M; King P J; Nayar R ; Wilkes S ; | Perceptions of Adolescent Patients of the "Lived Experience" of Type 1 Diabetes | 2017 | Design not quantitative |
| Klee P ; Bussien C ; Castellsague M ; Combescure C ; Dirlewanger M ; Girardin C ; Mando J L; Perrenoud L ; Salomon C ; Schneider F ; et al ; | Telemonitoring via a mobile device app reduces hba1c in type 1 diabetic children, without increasing the prevalence of hypoglycemia | 2018 | Design not quantitative |
| Klee P ; Bussien C ; Castellsague M ; Combescure C ; Dirlewanger M ; Girardin C ; Mando J L; Perrenoud L ; Salomon C ; Schneider F ; Schwitzgebel V M; | An Intervention by a Patient-Designed Do-It-Yourself Mobile Device App Reduces HbA1c in Children and Adolescents with Type 1 Diabetes: A Randomized Double-Crossover Study | 2018 | Relationship between hypoglycemia and outcome not assessed |
| Knight Madison F; | The effects of glucose levels on academic performance of children and adolescents with type 1 diabetes mellitus | 2018 | Outcomes were cognitive functioning |
| Kordonouri O ; Pankowska E ; Rami B ; Kapellen T ; Coutant R ; Hartmann R ; Lange K ; Knip M ; Danne T ; | Sensor-augmented pump therapy from the diagnosis of childhood type 1 diabetes: results of the Paediatric Onset Study (ONSET) after 12 months of treatment | 2010 | Relationship between hypoglycemia and outcome not assessed |
| Lang E G; King B R; Miller M N; Dunn S V; Price D A; Foskett D C; | Initiation of insulin pump therapy in children at diagnosis of type 1 diabetes resulted in improved long-term glycemic control | 2017 | Relationship between hypoglycemia and outcome not assessed |
| Lawrence J M; Standiford D A; Loots B ; Klingensmith G J; Williams D E; Ruggiero A ; Liese A D; Bell R A; Waitzfelder B E; McKeown R E; Study Search for Diabetes in Youth; | Prevalence and correlates of depressed mood among youth with diabetes: the SEARCH for Diabetes in Youth study | 2006 | Population not young people with type 1 diabetes |
| Lewis Kevin R; | Effectiveness of Continuous Glucose Monitoring in Children, Adolescents and Young Adults with Poorly Controlled Type 1 Diabetes | 2013 | Relationship between hypoglycemia and outcome not assessed |
| Lin A ; Northam E A; Rankins D ; Werther G A; Cameron F J; | Neuropsychological profiles of young people with type 1 diabetes 12 yr after disease onset | 2010 | Outcomes were cognitive functioning |
| Ly T T; Anderson M ; McNamara K A; Davis E A; Jones T W; | Neurocognitive outcomes in young adults with early-onset type 1 diabetes: a prospective follow-up study | 2011 | Outcomes were cognitive functioning |
| Ly T T; Nicholas J A; Retterath A ; Mun Lim ; E ; Davis E A; Jones T W; | Reduction of severe hypoglycemia with sensor-augmented insulin pump therapy and automated insulin suspension in patients with type 1 diabetes | 2013 | Relationship between hypoglycemia and outcome not assessed |
| Majidi S ; Driscoll K A; Raymond J K; | Anxiety in children and adolescents with type 1 diabetes | 2015 | Design not quantitative |
| Marcelino Daniela Botti; de Barros Carvalho ; Maria Dalva ; | Reflections on the Diabetes Type 1 and its Relation to the Emotional Aspect | 2005 | Not in English |
| Markowitz J T; Pratt K ; Aggarwal J ; Volkening L K; Laffel L M; | Psychosocial correlates of continuous glucose monitoring use in youth and adults with type 1 diabetes and parents of youth | 2012 | Relationship between hypoglycemia and outcome not assessed |
| Matyka K A; Crawford C ; Wiggs L ; Dunger D B; Stores G ; | Alterations in sleep physiology in young children with insulin-dependent diabetes mellitus: relationship to nocturnal hypoglycemia | 2000 | Other outcomes |
| Mayer-Davis E J; Maahs D M; Bishop F K; Driscoll K A; Hunter C M; Kichler J C; Thomas J M; Bishop F ; Bouffard A ; Clay M ; et al ; | Efficacy of the Flexible Lifestyles Empowering Change intervention on metabolic and psychosocial outcomes in adolescents with type 1 diabetes (FLEX): a randomised controlled trial | 2018 | Relationship between hypoglycemia and outcome not assessed |
| McCarthy A M; Lindgren S ; Mengeling M A; Tsalikian E ; Engvall J C; | Effects of diabetes on learning in children | 2002 | Outcomes were cognitive functioning |
| McMahon S K; Airey F L; Marangou D A; McElwee K J; Carne C L; Clarey A J; Davis E A; Jones T W; | Insulin pump therapy in children and adolescents: improvements in key parameters of diabetes management including quality of life | 2005 | Relationship between hypoglycemia and outcome not assessed |
| McNeilly A D; McCrimmon R J; | The Scylla and Charybdis of glucose control in childhood type 1 diabetes? | 2015 | Outcomes were cognitive functioning |
| Messenger Carla L; | Anxiety sensitivity in adolescents with type I diabetes and their parents: Relationship to the fear of hypoglycemia and prediction of metabolic control | 2006 | Design not quantitative |
| Miller Kellee M; | Assessment of the impact of attention deficit hyperactivity disorder on diabetes management and glycemic control among adolescents and young adults with type 1 diabetes | 2016 | Design not quantitative |
| Monzon A ; McDonough R ; Meltzer L J; Patton S R; | Sleep and type 1 diabetes in children and adolescents: Proposed theoretical model and clinical implications | 2019 | Design not quantitative |
| Moura da Cruz; DÃ©a Silvia ; de Lima Silva ; Kenya ; Batista de Souza; JosÃ© Tadeu ; Lima da NÃ³brega; Maria Miriam ; da Silva Reichert ; Altamira Pereira ; Marques Daniela Karina AntÃ£o; Collet Neusa ; | Experiences of adolescents with diabetes mellitus from the perspective of the ethics of alterity | 2018 | Design not quantitative |
| Mueller-Godeffroy E ; Vonthein R ; Ludwig-Seibold C ; Heidtmann B ; Boettcher C ; Kramer M ; Hessler N ; Hilgard D ; Lilienthal E ; Ziegler A ; Wagner V M; German Working Group for Pediatric Pump; Therapy ; | Psychosocial benefits of insulin pump therapy in children with diabetes type 1 and their families: The pumpkin multicenter randomized controlled trial | 2018 | Relationship between hypoglycemia and outcome not assessed |
| Muller-Godeffroy E ; Treichel S ; Wagner V M; German Working Group for Paediatric Pump; Therapy ; | Investigation of quality of life and family burden issues during insulin pump therapy in children with Type 1 diabetes mellitus--a large-scale multicentre pilot study | 2009 | Relationship between hypoglycemia and outcome not assessed |
| Murphy H R; Wadham C ; Hassler-Hurst J ; Rayman G ; Skinner T C; Families ; Adolescents Communication ; Teamwork Study ; Group ; | Randomized trial of a diabetes self-management education and family teamwork intervention in adolescents with Type 1 diabetes | 2012 | Relationship between hypoglycemia and outcome not assessed |
| Musen G ; Jacobson A M; Ryan C M; Cleary P A; Waberski B H; Weinger K ; Dahms W ; Bayless M ; Silvers N ; Harth J ; White N ; Diabetes Control ; Complications Trial ; Epidemiology of Diabetes, Interventions; Complications Research ; Group ; | Impact of diabetes and its treatment on cognitive function among adolescents who participated in the Diabetes Control and Complications Trial | 2008 | Outcomes were cognitive functioning |
| Naguib J M; Kulinskaya E ; Lomax C L; Garralda M E; | Neuro-cognitive performance in children with type 1 diabetes--a meta-analysis | 2009 | Outcomes were cognitive functioning |
| Nct ; | Psychosocial Issues in Insulin Pump Therapy in Children With Type 1 Diabetes Mellitus (DM) | 2011 | Relationship between hypoglycemia and outcome not assessed |
| Nordfeldt S ; Ludvigsson J ; | Fear and other disturbances of severe hypoglycaemia in children and adolescents with type 1 diabetes mellitus | 2005 | Population not young people with type 1 diabetes |
| Northam E ; Bowden S ; Anderson V ; Court J ; | Neuropsychological functioning in adolescents with diabetes | 1992 | Not published after 2000 |
| Northam E A; Matthews L K; Anderson P J; Cameron F J; Werther G A; | Psychiatric morbidity and health outcome in Type 1 diabetes--perspectives from a prospective longitudinal study | 2005 | Other outcomes |
| Northam E A; Anderson P J; Jacobs R ; Hughes M ; Warne G L; Werther G A; | Neuropsychological profiles of children with type 1 diabetes 6 years after disease onset | 2001 | Outcomes were cognitive functioning |
| Northam E A; Anderson P J; Werther G A; Warne G L; Andrewes D ; | Predictors of change in the neuropsychological profiles of children with type 1 diabetes 2 years after disease onset | 1999 | Not published after 2000 |
| Nuboer R ; Borsboom G J; Zoethout J A; Koot H M; Bruining J ; | Effects of insulin pump vs. injection treatment on quality of life and impact of disease in children with type 1 diabetes mellitus in a randomized, prospective comparison | 2008 | Relationship between hypoglycemia and outcome not assessed |
| Nyer Maren B; | Fear of hypoglycemia: Psychological associations and diabetes control in youth with type 1 diabetes and their parents | 2010 | Design not quantitative |
| Opipari-Arrigan L ; Fredericks E M; Burkhart N ; Dale L ; Hodge M ; Foster C ; | Continuous subcutaneous insulin infusion benefits quality of life in preschool-age children with type 1 diabetes mellitus | 2007 | Relationship between hypoglycemia and outcome not assessed |
| Pacaud D ; | Hypoglycemia: the Achilles heel of the treatment of children with type 1 diabetes | 2002 | Design not quantitative |
| Palmert M ; | A mobile app for the self-management of type 1 diabetes among adolescents: lessons learned from a randomized controlled trial | 2019 | Design not quantitative |
| Phillip M ; Danne T ; Shalitin S ; Buckingham B ; Laffel L ; Tamborlane W ; Battelino T ; Consensus Forum ; Participants ; | Use of continuous glucose monitoring in children and adolescents ( ) | 2012 | Design not quantitative |
| Pickup J C; Ford Holloway ; M ; Samsi K ; | Real-time continuous glucose monitoring in type 1 diabetes: a qualitative framework analysis of patient narratives | 2015 | Design not quantitative |
| Pillar G ; Schuscheim G ; Weiss R ; Malhotra A ; McCowen K C; Shlitner A ; Peled N ; Shehadeh N ; | Interactions between hypoglycemia and sleep architecture in children with type 1 diabetes mellitus | 2003 | Other outcomes |
| Prahalad P ; Tanenbaum M ; Hood K ; Maahs D M; | Diabetes technology: improving care, improving patient-reported outcomes and preventing complications in young people with Type 1 diabetes | 2018 | Design not quantitative |
| Price K J; Knowles J A; Fox M ; Wales J K; Heller S ; Eiser C ; Freeman J V; group K ICk-OFF Study; | Effectiveness of the Kids in Control of Food (KICk-OFF) structured education course for 11-16 year olds with Type 1 diabetes | 2016 | Relationship between hypoglycemia and outcome not assessed |
| Puczynski S ; Puczynski M S; Ryan C M; | Hypoglycemia in children with insulin-dependent diabetes mellitus | 1992 | Not published after 2000 |
| Pyatak E A; Sequeira P A; Vigen C L; Weigensberg M J; Wood J R; Montoya L ; Ruelas V ; Peters A L; | Clinical and Psychosocial Outcomes of a Structured Transition Program Among Young Adults With Type 1 Diabetes | 2017 | Population not young people with type 1 diabetes |
| Robling M ; McNamara R ; Bennert K ; Butler C C; Channon S ; Cohen D ; Crowne E ; Hambly H ; Hawthorne K ; Hood K ; Longo M ; Lowes L ; Pickles T ; Playle R ; Rollnick S ; Thomas-Jones E ; Gregory J W; | The effect of the Talking Diabetes consulting skills intervention on glycaemic control and quality of life in children with type 1 diabetes: cluster randomised controlled trial (DEPICTED study) | 2012 | Relationship between hypoglycemia and outcome not assessed |
| Rodrigues Vilela ; V ; de Castro Ruiz Marques; A ; Schamber C R; Bazotte R B; | Hypoglycemia induced by insulin as a triggering factor of cognitive deficit in diabetic children | 2014 | Outcomes were cognitive functioning |
| Rodriguez Perez ; C ; Lizondo Escuder ; A ; Lopez Garcia ; M J ; Escriva Cholbi ; L ; Alpera Lacruz ; R ; Collado Perez ; C ; | [A study of variability in glycaemia in children and adolescents with diabetes mellitus type 1 on treatment with insulin glargine] | 2008 | Not in English |
| Rousseau K ; | Children as young as 4 years of age with type 1 diabetes showed understanding and competence in managing their condition | 2007 | Design not quantitative |
| Rovet J F; Ehrlich R M; | The effect of hypoglycemic seizures on cognitive function in children with diabetes: a 7-year prospective study | 1999 | Not published after 2000 |
| Rubin R R; Peyrot M ; Group Star Study; | Health-related quality of life and treatment satisfaction in the Sensor-Augmented Pump Therapy for A1C Reduction 3 (STAR 3) trial | 2012 | Relationship between hypoglycemia and outcome not assessed |
| Ryan C M; | Neurobehavioral complications of type I diabetes. Examination of possible risk factors | 1988 | Not published after 2000 |
| Ryan Christopher M; | Does severe hypoglycaemia disrupt academic achievement in children with early onset diabetes? | 2012 | Design not quantitative |
| Sato E ; Ohsawa I ; Kataoka J ; Miwa M ; Tsukagoshi F ; Sato J ; Oshida Y ; Sato Y ; | Socio-psychological problems of patients with late adolescent onset type 1 diabetes--analysis by qualitative research | 2003 | Population not young people with type 1 diabetes |
| Schierloh U ; Aguayo G A; Fichelle M ; De Melo Dias ; C ; Celebic A ; Vaillant M T; De Beaufort ; C ; | Does predicted low suspend pump treatment improve control and quality of sleep in children with type 1 diabetes and their caregiver? the quest study | 2018 | Relationship between hypoglycemia and outcome not assessed |
| Schwartz D D; Wasserman R ; Powell P W; Axelrad M E; | Neurocognitive outcomes in pediatric diabetes: a developmental perspective | 2014 | Outcomes were cognitive functioning |
| Semenkovich K ; Patel P P; Pollock A B; Beach K A; Nelson S ; Masterson J J; Hershey T ; Arbelaez A M; | Academic abilities and glycaemic control in children and young people with Type 1 diabetes mellitus | 2016 | Outcomes were cognitive functioning |
| Shehata Ghaydaa ; Eltayeb Azza ; | Cognitive function and event-related potentials in children with type 1 diabetes mellitus | 2010 | outcomes were cognitive functioning |
| Sherif E M; El Tonbary ; K Y ; Abd Aziz ; M M ; | Comparative study between the use of insulin glargine and intermediate acting insulin (NPH) in type 1 diabetic children less than eight years old | 2014 | Relationship between hypoglycemia and outcome not assessed |
| Soltesz G ; | Hypoglycaemia in the diabetic child | 1993 | Not published after 2000 |
| Sullivan-Bolyai Susan ; Crawford Sybil ; Johnson Kim ; Ramchandani Neesha ; Quinn Diane ; Dâ€™Alesandro Bianca ; Stern Kailyn ; Lipman Terry ; Melkus Gail ; Streisand Randi ; | PREP-T1 (Preteen Re-Education With Parentsâ€“Type 1 Diabetes) Feasibility Intervention Results | 2016 | Relationship between hypoglycemia and outcome not assessed |
| Tamborlane W V; Swan K ; Sikes K A; Steffen A T; Weinzimer S A; | The renaissance of insulin pump treatment in childhood type 1 diabetes | 2006 | Design not quantitative |
| Tansey M ; Laffel L ; Cheng J ; Beck R ; Coffey J ; Huang E ; Kollman C ; Lawrence J ; Lee J ; Ruedy K ; Tamborlane W ; Wysocki T ; Xing D ; Juvenile Diabetes Research Foundation Continuous Glucose Monitoring; Group ; | Satisfaction with continuous glucose monitoring in adults and youths with Type 1 diabetes | 2011 | Relationship between hypoglycemia and outcome not assessed |
| Tiberg I ; HallstrÃ¶m I ; JÃ¶nsson L ; Carlsson A ; | Comparison of hospital-based and hospital-based home care at diabetes onset in children | 2014 | Relationship between hypoglycemia and outcome not assessed |
| von Sengbusch ; S ; Muller-Godeffroy E ; Hager S ; Reintjes R ; Hiort O ; Wagner V ; | Mobile diabetes education and care: intervention for children and young people with Type 1 diabetes in rural areas of northern Germany | 2006 | Relationship between hypoglycemia and outcome not assessed |
| Waller H ; Eiser C ; Knowles J ; Rogers N ; Wharmby S ; Heller S ; Hall C ; Greenhalgh S ; Tinklin T ; Metcalfe C ; Millard E ; Parkin V ; Denial M ; Price K ; | Pilot study of a novel educational programme for 11-16 year olds with type 1 diabetes mellitus: the KICk-OFF course | 2008 | Relationship between hypoglycemia and outcome not assessed |
| Wdowik M J; Kendall P A; Harris M A; | College students with diabetes: using focus groups and interviews to determine psychosocial issues and barriers to control | 1997 | Not published after 2000 |
| Weinzimer S A; Doyle E A; Steffen A T; Sikes K A; Tamborlane W V; | Rediscovery of insulin pump treatment of childhood type 1 diabetes | 2004 | Design not quantitative |
| Weissberg-Benchell J ; Hessler D ; Polonsky W H; Fisher L ; | Psychosocial Impact of the Bionic Pancreas During Summer Camp | 2016 | Relationship between hypoglycemia and outcome not assessed |
| Weitzman E R; Kelemen S ; Quinn M ; Eggleston E M; Mandl K D; | Participatory surveillance of hypoglycemia and harms in an online social network | 2013 | Population not young people with type 1 diabetes |
| Wilson D M; Buckingham B A; Kunselman E L; Sullivan M M; Paguntalan H U; Gitelman S E; | A two-center randomized controlled feasibility trial of insulin pump therapy in young children with diabetes | 2005 | Relationship between hypoglycemia and outcome not assessed |
| Wilson V ; | Students' experiences of managing type 1 diabetes... includes discussion | 2010 | Design not quantitative |
| Wysocki Tim ; | Behavioral Assessment and Intervention in Pediatric Diabetes | 2006 | Design not quantitative |
| Wysocki T ; Harris M A; Mauras N ; Fox L ; Taylor A ; Jackson S C; White N H; Wysocki Tim ; Harris Michael A; Mauras Nelly ; Fox Larry ; Taylor Alexandra ; Jackson S Craig; White Neil H; | Absence of adverse effects of severe hypoglycemia on cognitive function in school-aged children with diabetes over 18 months | 2003 | Outcomes were cognitive functioning |
| Ye C Y; Jeppson T C; Kleinmaus E M; Kliems H M; Schopp J M; Cox E D; | Outcomes That Matter to Teens With Type 1 Diabetes | 2017 | Design not quantitative |
| Zeitler Phil ; Fu Junfen ; Tandon Nikhil ; Nadeau Kristen ; Urakami Tatsuhiko ; Barrett Timothy ; Maahs David ; | Type 2 diabetes in the child and adolescent | 2014 | Population not young people with type 1 diabetes |
| Zennaki A ; Niar S ; Naceur M ; Aichaoui H ; Ouzzaa K ; Aoui A ; Amari Z ; Reguieg A ; Gharnouti M ; Bessahraoui M ; et al ; | Effect of paramedical treatment codified on balance, quality of life and knowledge of teenagers suffering from T1DM persisting imbalance | 2015 | Relationship between hypoglycemia and outcome not assessed |
| Alderson P ; Sutcliffe K ; Curtis K ; | Children as partners with adults in their medical care | 2006 | Design not quantitative |
| Amillategui Blanca ; Mora Epifanio ; Calle Jose Ramon; Giralt Patricio ; | Special needs of children with type 1 diabetes at primary school: perceptions from parents, children, and teachers | 2009 | Relationship between hypoglycemia and outcome not assessed |
| Barnard Katharine ; James Janet ; Kerr David ; Adolfsson Peter ; Runion Asher ; Serbedzija George ; | Impact of Chronic Sleep Disturbance for People Living With T1 Diabetes | 2016 | Population not young people with type 1 diabetes |
| Bomba Franziska ; Muller-Godeffroy Esther ; von Sengbusch ; Simone ; | Experiences in Sensor-Augmented Pump Therapy in Families with two Children with Type 1 diabetes: A Qualitative Study | 2018 | Design not quantitative |
| Chiarelli F ; Verrotti A ; di Ricco ; L ; Altobelli E ; Morgese G ; | Hypoglycaemic symptoms described by diabetic children and their parents | 1998 | Not published after 2000 |
| Dall'Antonia C ; Zanetti M L; | [Insulin self administration in children with diabetes mellitus, type 1] | 2000 | Not in English |
| Law J R; Yesiltepe-Mutlu G ; Helms S ; Meyer E ; Ozsu E ; Cizmecioglu F ; Lin F C; Hatun S ; Calikoglu A S; | Adolescents with Type 1 diabetes mellitus experience psychosensorial symptoms during hypoglycaemia | 2014 | Other outcomes |
| Marrero D G; Guare J C; Vandagriff J L; Fineberg N S; | Fear of hypoglycemia in the parents of children and adolescents with diabetes: maladaptive or healthy response? | 1997 | Not published after 2000 |
| McCarthy Ann Marie; Lindgren Scott ; Mengeling Michelle A; Tsalikian Eva ; Engvall Janet ; | Factors associated with academic achievement in children with type 1 diabetes | 2003 | Population not young people with type 1 diabetes |
| Moore Susan M; Hackworth Naomi J; Hamilton Victoria E; Northam Elisabeth P; Cameron Fergus J; | Adolescents with type 1 diabetes: parental perceptions of child health and family functioning and their relationship to adolescent metabolic control | 2013 | Relationship between hypoglycemia and outcome not assessed |
| Noser Amy E; Majidi Shideh ; Finch Jonathan ; Clements Mark A; Youngkin Erin M; Patton Susana R; | Authoritarian parenting style predicts poorer glycemic control in children with new-onset type 1 diabetes | 2018 | Population not young people with type 1 diabetes |
| Olinder Anna Lindholm; Nyhlin Kerstin Ternulf; Smide Bibbi ; | Clarifying responsibility for self-management of diabetes in adolescents using insulin pumps--a qualitative study | 2011 | Design not quantitative |
| Pyatak Elizabeth A; Florindez Daniella ; Peters Anne L; Weigensberg Marc J; | "We are all gonna get diabetic these days": the impact of a living legacy of type 2 diabetes on Hispanic young adults' diabetes care | 2014 | Population not young people with type 1 diabetes |
| Rothman Russell L; Mulvaney Shelagh ; Elasy Tom A; VanderWoude Ann ; Gebretsadik Tebeb ; Shintani Ayumi ; Potter Amy ; Russell William E; Schlundt David ; | Self-management behaviors, racial disparities, and glycemic control among adolescents with type 2 diabetes | 2008 | Population not young people with type 1 diabetes |
| Schoenle E J; Schoenle D ; Molinari L ; Largo R H; | Impaired intellectual development in children with Type I diabetes: association with HbA(1c), age at diagnosis and sex | 2002 | Outcomes were cognitive functioning |
| Shield J P; Baum J D; | Complications of diabetes in childhood | 1993 | Not published after 2000 |
| Turner Sara L; Berg Cynthia A; Butner Jonathan E; Wiebe Deborah J; | Attention Problems as a Predictor of Type 1 Diabetes Adherence and Metabolic Control Across Adolescence | 2018 | No exposure of hypoglycemia |
| Altobelli E ; Valenti M ; Verrotti A ; Masedu F ; Tiberti S ; Chiarelli F ; Di Orio ; F ; | Family and disease management in young type 1 diabetic patients | 2000 | Relationship between hypoglycemia and outcome not assessed |
| Anonymous ; | Effects of intensive diabetes therapy on neuropsychological function in adults in the Diabetes Control and Complications Trial | 1996 | Not published after 2000 |
| Banion C R; Miles M S; Carter M C; | Problems of mothers in management of children with diabetes | 1983 | Not published after 2000 |
| Cooper Matthew N; McNamara Kaitrin A. R; de Klerk ; Nicholas H ; Davis Elizabeth A; Jones Timothy W; | School performance in children with type 1 diabetes: a contemporary population-based study | 2016 | Outcomes were cognitive functioning |
| Di Battista ; Ashley M ; Hart Trevor A; Greco Laurie ; Gloizer Jan ; | Type 1 diabetes among adolescents: reduced diabetes self-care caused by social fear and fear of hypoglycemia | 2009 | Relationship between hypoglycemia and outcome not assessed |
| Dumont R H; Jacobson A M; Cole C ; Hauser S T; Wolfsdorf J I; Willett J B; Milley J E; Wertlieb D ; | Psychosocial predictors of acute complications of diabetes in youth | 1995 | Not published after 2000 |
| Haugbolle Lotte Stig; Devantier Kristina ; Frydenlund Bente ; | A user perspective on type 1 diabetes: sense of illness, search for freedom and the role of the pharmacy | 2002 | Design not quantitative |
| Hilliard Marisa E; Holmes Clarissa S; Chen Rusan ; Maher Kathryn ; Robinson Elizabeth ; Streisand Randi ; | Disentangling the roles of parental monitoring and family conflict in adolescents' management of type 1 diabetes | 2013 | No exposure of hypoglycemia |
| Jacobson A M; Hauser S T; Lavori P ; Willett J B; Cole C F; Wolfsdorf J I; Dumont R H; Wertlieb D ; | Family environment and glycemic control: a four-year prospective study of children and adolescents with insulin-dependent diabetes mellitus | 1994 | Not published after 2000. |
| Jarosz-Chobot P ; Guthrie D W; Otto-Buczkowska E ; Koehler B ; | Self-care of young diabetics in practice | 2000 | Design not quantitative |
| La Greca ; A M ; Auslander W F; Greco P ; Spetter D ; Fisher E B; Jr ; Santiago J V; | I get by with a little help from my family and friends: adolescents' support for diabetes care | 1995 | Not published after 2000 |
| Lin Ashleigh ; Northam Elisabeth A; Werther George A; Cameron Fergus J; | Risk factors for decline in IQ in youth with type 1 diabetes over the 12 years from diagnosis/illness onset | 2015 | Outcomes were cognitive functioning |
| Lin Shih-Yi ; Lin Cheng-Li ; Hsu Wu-Huei ; Lin Cheng-Chieh ; Fu Yun-Ching ; | Association of attention deficit hyperactivity disorder with recurrent hypoglycemia in type 1 diabetes mellitus | 2019 | Other outcomes |
| Marshall Katarzyna ; Martin Halley ; Siarkowski Amer ; Kim ; | Exploring Perceptions about Insulin Dependent Diabetes Mellitus in Adolescent Patients and Peers | 2018 | Design not quantitative |
| Meunier J ; Dorchy H ; Luminet O ; | Does family cohesiveness and parental alexithymia predict glycaemic control in children and adolescents with diabetes? | 2008 | Population not young people with type 1 diabetes |
| Newbould J ; Francis S A; Smith F ; | Young people's experiences of managing asthma and diabetes at school | 2007 | Design not quantitative |
| Patton Susana R; Dolan Lawrence M; Henry Racquel ; Powers Scott W; | Parental fear of hypoglycemia: young children treated with continuous subcutaneous insulin infusion | 2007 | Design not quantitative |
| Seiffge-Krenke Inge ; Laursen Brett ; Dickson Daniel J; Hartl Amy C; | Declining metabolic control and decreasing parental support among families with adolescents with diabetes: the risk of restrictiveness | 2013 | No exposure of hypoglycemia |
| Silverstein J H; Johnson S ; | Psychosocial challenge of diabetes and the development of a continuum of care | 1994 | Not published after 2000 |
| Smith Charlotte B; Choudhary Pratik; Pernet Andrew; Hopkins David; Amiel Stephanie A; | Hypoglycemia unawareness is associated with reduced adherence to therapeutic decisions in patients with type 1 diabetes: evidence from a clinical audit | 2009 | Relationship between hypoglycemia and outcome not assessed |
| Spencer J E; Cooper H C; Milton B ; | The lived experiences of young people (13-16 years) with Type 1 diabetes mellitus and their parents--a qualitative phenomenological study | 2013 | Design not quantitative |
| Strand Marianne ; Brostrom Anders ; Haugstvedt Anne ; | Adolescents' perceptions of the transition process from parental management to self-management of type 1 diabetes | 2019 | Design not quantitative |
| Vyas S ; Mullee M A; Kinmonth A L; | British Diabetic Association holidays--what are they worth? | 1988 | Not published after 2000 |
| Wu Yelena P; Graves Montserrat M; Roberts Michael C; Mitchell Adela C; | Is insulin pump therapy better than injection for adolescents with diabetes? | 2010 | No exposure of hypoglycemia |
| Yokota Ichiro ; Amemiya Shin ; Kida Kaichi ; Sasaki Nozomu ; Matsuura Nobuo ; Japanese Study Group of Insulin Therapy for; Childhood ; Adolescent Diabetes ; | Past 10-year status of insulin therapy for preschool-age Japanese children with type 1 diabetes | 2005 | Population not young people with type 1 diabetes |
| Cecilia-Costa R, Volkening LK, Laffel LM | Factors associated with disordered eating behaviours in adolescents with Type 1 diabetes | 2019 | No exposure of hypoglycemia |
| Cobry EC, Hamburger E, Jaser SS, Cobry E, Jaser S | Impact of the Hybrid Closed-Loop System on Sleep and Quality of Life in Youth with Type 1 Diabetes and Their Parents | 2019 | No exposure of hypoglycemia |
| Covener Ozcelik C, Aktas E, Sen Celasin N, Karahan Okuroglu G, Sahin S | The Development and Validation of a Turkish Insulin Treatment Self-management Scale Child Form (Ages 8-18) and Parent Form | 2019 | No exposure of hypoglycemia |
| Fleming M, Fitton CA, Steiner MFC, McLay JS, Clark D, King A, et al | Educational and Health Outcomes of Children Treated for Type 1 Diabetes: Scotland-Wide Record Linkage Study of 766,047 Children | 2019 | No exposure of hypoglycemia |
| Gurkan KP, Bahar Z, Bober E | Effects of a home-based nursing intervention programme among adolescents with type 1 diabetes | 2019 | No exposure of hypoglycemia |
| Jaser SS, Bergner EM, Hamburger ER, Bhatia S, Lyttle M, Bell GE, et al | Pilot trial of a sleep-promoting intervention for children with type 1 diabetes | 2021 | No exposure of hypoglycemia |
| Kane NS, Hoogendoorn CJ, Commissariat PV, Schulder TE, Gonzalez JS | Glycemic control and self-rated health among ethnically diverse adolescents with type 1 diabetes. | 2020 | No exposure of hypoglycemia |
| Pirie FJ, Jairam V, Paruk IM, Connolly C, Motala AA | High frequency of hypoglycaemia in patients with type 1 diabetes mellitus attending a tertiary diabetes clinic in Durban, South Africa | 2019 | Population not young people with type 1 diabetes |
| Galler A, Hilgard D, Bollow E, Hermann T, Kretschmer N, Maier B, et al. | Psychological care in children and adolescents with type 1 diabetes in a real‐world setting and associations with metabolic control | 2020 | Other outcomes |
| Griggs S, Redeker NS, Jeon S, Grey M | Daily variations in sleep and glucose in adolescents with type 1 diabetes | 2020 | Other outcomes |
| Knight MF, Perfect MM. | Glycemic control influences on academic performance in youth with Type 1 diabetes | 2019 | Other outcomes |
| Shapiro ALB, Dabelea D, Stafford JM, D'Agostino R, Jr., Pihoker C, Liese AD, et al | Cognitive Function in Adolescents and Young Adults With Youth-Onset Type 1 Versus Type 2 Diabetes: The SEARCH for Diabetes in Youth Study | 2021 | Other outcomes |
| Hitt TA, Smith J, Forth EL, Garren P, Olivos-Stewart D, La Vega MD, et al. | 1292-P: Continuous Glucose Monitor Use Protects against the Negative Impact of Fear of Hypoglycemia on Sleep Parameters in Adolescents with Type 1 Diabetes | 2021 | Design not quantitative |
| Beato-Víbora PI, Gallego-Gamero F, Lázaro-Martín L, Romero-Pérez MdM, Arroyo-Díez FJ | . Prospective Analysis of the Impact of Commercialized Hybrid Closed-Loop System on Glycemic Control, Glycemic Variability, and Patient-Related Outcomes in Children and Adults: A Focus on Superiority Over Predictive Low-Glucose Suspend Technology | 2020 | Relationship between hypoglycemia and outcome not assessed |
| Berget C, Messer LH, Vigers T, Frohnert BI, Pyle L, Wadwa RP, et al. | Six months of hybrid closed loop in the real-world: An evaluation of children and young adults using the 670G system | 2020 | Relationship between hypoglycemia and outcome not assessed |
| Blair JC, McKay A, Ridyard C, Thornborough K, Bedson E, Peak M, et al. | Continuous subcutaneous insulin infusion versus multiple daily injection regimens in children and young people at diagnosis of type 1 diabetes: pragmatic randomised controlled trial and economic evaluation | 2019 | Relationship between hypoglycemia and outcome not assessed |
| Braune K, Boss K, Schmidt-Herzel J, Gajewska KA, Thieffry A, Schulze L, et al. | Shaping Workflows in Digital and Remote Diabetes Care During the COVID-19 Pandemic via Service Design: Prospective, Longitudinal, Open-label Feasibility Trial | 2021 | Relationship between hypoglycemia and outcome not assessed |
| Chatzakis C, Floros D, Papagianni M, Tsiroukidou K, Kosta K, Vamvakis A, et al. | The Beneficial Effect of the Mobile Application in Children and Adolescents with Type 1 Diabetes Mellitus: A Randomized Controlled Trial | 2019 | Relationship between hypoglycemia and outcome not assessed |
| Forlenza GP, Wadwa RP, Messer LH, Ekhlaspour L, Maahs DM, Town M, et al | Successful At-Home Use of the Tandem Control-IQ Artificial Pancreas System in Young Children During a Randomized Controlled Trial | 2019 | Relationship between hypoglycemia and outcome not assessed |
| Halbron M, Bourron O, Andreelli F, Ciangura C, Jacqueminet S, Popelier M, et al. | Insulin Pump Combined with Flash Glucose Monitoring: A Therapeutic Option to Improve Glycemic Control in Severely Nonadherent Patients with Type 1 Diabetes | 2019 | Relationship between hypoglycemia and outcome not assessed |
| Laffel LM, Kanapka LG, Beck RW, Bergamo K, Clements MA, Criego A, et al | Effect of Continuous Glucose Monitoring on Glycemic Control in Adolescents and Young Adults With Type 1 Diabetes: A Randomized Clinical Trial | 2020 | Relationship between hypoglycemia and outcome not assessed |
| Lim STJ, Huang F, Lek N, Pereira K | Flash Continuous Home Glucose Monitoring to Improve Adherence to Self-Monitoring of Blood Glucose and Self-Efficacy in Adolescents With Type 1 Diabetes. | 2020 | Relationship between hypoglycemia and outcome not assessed |
| Markosyan R, Perikhanyan A | Health-Related Quality of Life in Children with Type 1 Diabetes in Armenia | 2019 | Relationship between hypoglycemia and outcome not assessed |
| Messaaoui A, Tenoutasse S, Crenier L. | Flash Glucose Monitoring Accepted in Daily Life of Children and Adolescents with Type 1 Diabetes and Reduction of Severe Hypoglycemia in Real-Life Use | 2019 | Relationship between hypoglycemia and outcome not assessed |
| Petruzelkova L, Jiranova P, Soupal J, Kozak M, Plachy L, Neuman V, et al. | Pre-school and school-aged children benefit from the switch from a sensor-augmented pump to an AndroidAPS hybrid closed loop: A retrospective analysis | 2021 | Relationship between hypoglycemia and outcome not assessed |
| Roberts AJ, Taplin CE, Isom S, Divers J, Saydah S, Jensen ET, et al. | Association between fear of hypoglycemia and physical activity in youth with type 1 diabetes: The SEARCH for diabetes in youth study | 2020 | Relationship between hypoglycemia and outcome not assessed |
| Verbeeten KC, Perez Trejo ME, Tang K, Chan J, Courtney JM, Bradley BJ, et al. | Fear of hypoglycemia in children with type 1 diabetes and their parents: Effect of pump therapy and continuous glucose monitoring with option of low glucose suspend in the CGM TIME trial | 2021 | Relationship between hypoglycemia and outcome not assessed |
| Berger G, Waldhoer T, Barrientos I, Kunkel D, Rami‐Merhar BM, Schober E, et al. | Association of insulin‐manipulation and psychiatric disorders: A systematic epidemiological evaluation of adolescents with type 1 diabetes in Austria | 2019 | Other outcomes |
