# Supplementary material for: The impact of hypoglycemia on quality of life and related outcomes in children and adolescents with type 1 diabetes: A systematic review
Source: PLoS One. 2021 Dec 2;16(12):e0260896. doi: 10.1371/journal.pone.0260896 (PMC8638919; doi:10.1371/journal.pone.0260896)
Supplement: S2 Table — (DOCX) [file pone.0260896.s004.docx]

**Table S2**. Overview of scales being used across studies

| **Instrument name** | **Aim** | **Number of items** | **Subscales** | **Recall period** | **Validated for use in children/adolescents with type 1 diabetes** |
| --- | --- | --- | --- | --- | --- |
| Adolescent Sleep/Wake scale | To measure overall subjective sleep quality | 28 | - Difficulty going to bed | 1 month | No |
|  |  |  | - Falling asleep |  |  |
|  |  |  | - Maintaining sleep |  |  |
|  |  |  | - Reinitiating sleep |  |  |
|  |  |  | - Returning to wakefulness |  |  |
| Center for Epidemiological Studies-Depression Scale | To assess self-reported depressive symptomatology | 20 | - N/A | 1 week | No |
| Child Posttraumatic Stress Reaction Index | To assess reactions after traumatic events | 20 | - N/A | N/A | No |
| Children's Depression Inventory- Short version | To screen for depression in medically ill children | 10 | - N/A | 2 weeks | Yes |
| Children’s  Hypoglycemia Index | To measure fear of hypoglycemia in children | 24 | - Situation | N/A | Yes |
|  |  |  | - General |  |  |
|  |  |  | - Behavior |  |  |
| Diabetes Quality of Life for Youth | To assess diabetes-specific health related quality of life | 52 | - Impact scale | N/A | Yes |
|  |  |  | - Life satisfaction scale |  |  |
|  |  |  | - Worries about diabetes |  |  |
|  |  |  | - Health perception |  |  |
| Diabetes Quality of Life for Youth Short Form | To assess diabetes-specific health related quality of life | 22 | - Impact of treatment | N/A | Yes |
|  |  |  | - Symptom impact |  |  |
|  |  |  | - Impact on activities |  |  |
|  |  |  | - Parents |  |  |
|  |  |  | - Worry |  |  |
|  |  |  | - Satisfaction |  |  |
| DISABKIDS DCGM-12 | To assess health related quality of life of children and adolescents with chronic medical conditions | 12 | - N/A | 4 weeks | Yes |
| DISABKIDS Diabetes Module | To assess diabetes-specific aspects of health-related quality of life | 10 | - Impact | 4 weeks | Yes |
|  |  |  | - Treatment |  |  |
| Diabetes Eating Problem Survey-Revised | To assess diabetes-specific self-reported disordered eating behaviors | 16 | - N/A | N/A | Yes |
| European Quality of life - 5 Dimensions (EQ-5D) - VAS scale | To evaluate general health status | 1 | - N/A | N/A | Yes |
| Hypoglycemia Fear Survey – Child version | To assess the levels of fear related to hypoglycemia | 25 | - Worries about hypoglycaemia | N/A | Yes |
|  |  |  | - Fear of hypoglycaemia related behaviors |  |  |
| KINDL-R | To assess health-related quality of life | 24/30 | - Physical wellbeing | 1 week | No |
|  |  |  | - Emotional wellbeing |  |  |
|  |  |  | - Family |  |  |
|  |  |  | - Friends |  |  |
|  |  |  | - Self-esteem |  |  |
|  |  |  | - Chronic illness scale |  |  |
| KIDSCREEN 10 index | To assess general health related quality of life | 10/11 | - Global QoL | 1 week | No |
| KIDSCREEN 27 | To measure subjective health and well-being | 27 | - Physical wellbeing | 1 week | No |
|  |  |  | - Psychological well-being |  |  |
|  |  |  | - Autonomy and relationships with parents |  |  |
|  |  |  | - School |  |  |
|  |  |  | - Relationships with friends or peers |  |  |
| Pediatric Quality of Life Inventory- Diabetes Module | To assess diabetes-specific health related quality of life | 32/33 | - Diabetes symptoms | 1 month | Yes |
|  |  |  | - Diabetes management |  |  |
| Pediatric Quality of Life Inventory – Generic Module | To assess health-related quality of life | 23 | - Physical functioning | 1 month | Yes |
|  |  |  | - Emotional functioning |  |  |
|  |  |  | - School functioning |  |  |
|  |  |  | - Social functioning |  |  |
| Screen for Child Anxiety-Related Disorders | To screen for signs of anxiety disorders in children | 41 | - Panic disorder / somatic symptoms | 3 months | No |
|  |  |  | - Generalized anxiety disorder |  |  |
|  |  |  | - Separation anxiety disorder |  |  |
|  |  |  | - Social anxiety disorder |  |  |
|  |  |  | - Significant school avoidance |  |  |
| State-Trait Anxiety Inventory for Children | To assess state and trait anxiety for children | 40 | - Trait Subscale | N/A | No |
|  |  |  | - State subscale |  |  |
