# Supplementary material for: The impact of hypoglycemia on quality of life and related outcomes in children and adolescents with type 1 diabetes: A systematic review
Source: PLoS One. 2021 Dec 2;16(12):e0260896. doi: 10.1371/journal.pone.0260896 (PMC8638919; doi:10.1371/journal.pone.0260896)
Supplement: S3 Table — (DOCX) [file pone.0260896.s005.docx]

**Table S3.** Quality assessment of the included studies

| **Author, year** | **Inclusion criteria** | **Subjects and setting** | **Exposure measured** | **Objective measure of condition** | **Confounders identified** | **Dealt with confounders** | **Outcomes measured valid** | **Appropriate analysis** |
| --- | --- | --- | --- | --- | --- | --- | --- | --- |
| Adler et al. (2017) [34] | Yes | Yes | Yes | Yes | Yes | No | Unclear | Yes |
| Al Hayek et al. (2014) [35] | Yes | Yes | No | Yes | Yes | Yes | Unclear | Unclear |
| Amiri et al. (2014) [38] | Yes | Yes | Unclear | Yes | Yes | Yes | Yes | Yes |
| Caferoğlu et al. (2016) [16] | Yes | Yes | Yes | Yes | Yes | Yes | Yes | Yes |
| Coolen et al. (2021) | Yes | Yes | Unclear | Yes | Yes | Yes | Yes | Yes |
| Dłużniak-Gołaska et al. (2019) [42] | Yes | Yes | Yes | Yes | Yes | Yes | Yes | Yes |
| Galler et al. (2021) | Yes | Yes | Yes | Yes | Yes | Yes | Yes | Yes |
| Gonder- Frederick et al. (2006) [37] | Yes | Unclear | Unclear | Yes | Yes | Yes | Yes | Yes |
| Hanberger et al. (2009) [27] | Unclear | Yes | No | Yes | Yes | Yes | Unclear | Yes |
| Hassan et al. (2017) [32] | Yes | Unclear | Unclear | Yes | Yes | No | Yes | No |
| Hoey et al. (2001) [36] | Yes | Yes | Yes | Yes | Yes | Unclear | Yes | No |
| Johnson et al. (2013) [15] | Yes | Yes | Yes | Yes | Yes | Yes | Yes | Yes |
| Jurgen et al. (2020) | Yes | Yes | Yes | Yes | Yes | No | Yes | Yes |
| Kalvya et al. (2011) [28] | Yes | Unclear | Yes | Yes | Yes | Yes | Yes | Yes |
| Lawrence et al. (2012) [41] | Yes | Yes | No | Yes | Yes | Yes | Yes | Yes |
| Matziou et al. (2010) [31] | Yes | Yes | Yes | Yes | Yes | No | Yes | Yes |
| Murillo et al. (2017) [14] | Yes | Yes | Yes | Yes | Yes | No | Unclear | No |
| Naugthon et al. (2008) [30] | Yes | Yes | No | Yes | Yes | Yes | Yes | Yes |
| Nip et al. (2019) [46] | Yes | Yes | No | Yes | Yes | No | Yes | Yes |
| Plener et al. (2015) [45] | Unclear | Yes | Yes | Yes | Yes | Yes | Yes | Yes |
| Riaz et al. (2017) [40] | Yes | Unclear | No | Yes | Yes | Yes | Unclear | No |
| Serkel-Schrama et al. (2016) [29] | Yes | Unclear | No | No | Yes | No | Yes | Yes |
| Shepard et al. (2014) [39] | Yes | Yes | Unclear | No | Yes | No | Yes | Yes |
| Sismanlar et al. (2012) [43] | Unclear | Yes | Yes | Yes | Yes | Yes | Unclear | Yes |
| Stahl-Pehe et al. (2013) [13] | Yes | Yes | No | Yes | Yes | Yes | Unclear | Yes |
| Strudwick et al. (2005) [44] | Yes | Unclear | Yes | Yes | Yes | Yes | Yes | Yes |
| Wagner et al. (2005) [33] | Yes | Unclear | Yes | Yes | Yes | Yes | Unclear | Yes |

Y, Yes; N, No; U, Unclear

Note. The option “unclear” was also used in case of mixed results. The option “unclear” was also used in case of mixed results. To assess whether a paper measured the outcome in a valid and reliable way, it was only coded “yes” if the included measurements were validated among adolescents with diabetes.
